# Implementation of study results in guidelines and adherence to guidelines in clinical practice

**DOI:** 10.3205/cto000128

**Published:** 2016-12-15

**Authors:** Frank Waldfahrer

**Affiliations:** 1 Department of Otolaryngology, Head & Neck Surgery, University Hospital of Erlangen, Germany

**Keywords:** guidelines, implementation, study results, adherence

## Abstract

Guidelines were introduced in hospital- and practice-based otorhinolaryngology in the 1990ies, and have been undergoing further development ever since. There are currently 20 guidelines on file at the German Society of Oto-Rhino-Laryngology, Head & Neck Surgery. The society has cooperated in further 34 guidelines. The quality of the guidelines has been continuously improved by concrete specifications put forward by the Association of the Scientific Medical Societies in Germany (Arbeitsgemeinschaft der Wissenschaftlichen Medizinischen Fachgesellschaften e.V., AWMF). Since increasing digitalization has made access to scientific publications quicker and simpler, relevant study results can be incorporated in guidelines more easily today than in the analog world. S2e and S3 guidelines must be based on a formal literature search with subsequent evaluation of the evidence. The consensus procedure for S2k guidelines is also regulated. However, the implementation of guidelines in routine medical practice must still be considered inadequate, and there is still a considerable need for improvement in adherence to these guidelines.

## 1 Introduction

What is thought is not always said; 

what is said is not always heard; 

what is heard is not always understood; 

what is understood is not always agreed; 

what is agreed is not always done; 

what is done is not always done again.

This aphorism of the zoologist and behaviorist Konrad Lorenz (1903–1989) is often cited in the context of communication psychology when the sender-receiver model of Shannon and Weaver is presented. 

Slightly modified, the general ideas of this citation can be transferred to the implementation of guidelines and study results into daily clinical practice because also hereby numerous possibilities of errors exist.

- If someone does not know that guidelines exist for specific clinical questions, he will not look for them and consequently not observe them.
- If someone does not know where those specific guidelines – simple and up-to-date – can be found, he will not take into consideration to learn about them.
- If someone knows about the existence of specific guidelines, he might be discouraged by technical problems (download only after registration; fee-based contents) or by an extent that cannot be managed in the daily routine and so does not further care for the content of the guidelines.
- Even if someone dealt with the content of the guidelines, he will not apply them concretely when the guideline has only low reference to everyday life and/or economic restrictions impede guideline-based therapy.
- Someone who has applied a guideline in an individual case, will not automatically adhere to this behavior in similar contexts.
- Someone who has applied a guideline in an individual case, will not automatically go through the whole process again from the search to the application.

## 2 The very beginning – historical aspects

The development of scientific guidelines in Germany is closely related with the AWMF, i.e. the Association of the Scientific Medical Societies in Germany [33]. Initiated by the Germany Society of Surgery, the AWMF was founded already in 1962. At that time, the German Society of Oto-Rhino-Laryngology, Head and Neck Surgery was called German Society of Otolaryngologists and was represented by Prof. Otto Erich Riecker (1910–1982), Head of the Department of Otolaryngology of the Hospital of Wuppertal from 1951 to 1976.

The fate of the AWMF in the following years was even more closely related with another otolaryngologist. From 1977 to 1990, Prof. Karl-Heinz Vosteen (1925–2009) held the position of secretary and from 1981 to 1985 he was vice-president of the society. From 1985 to 1991, he was president of the AWMF. Afterwards he was member of the guideline commission. 

During Vosteen’s presidency, the first activities to develop national guidelines were started, of course also ENT-specific issues were taken into consideration. The initiative was taken by the Advisory Council for Concerted Action in Healthcare (Sachverständigenrat für die konzertierte Aktion im Gesundheitswesen, SVRKAiG, developed from the Healthcare Reform Act in 1988; since 2003 called Advisory Council for Assessment of the Development in Healthcare). In the Healthcare Structure Reform Act (Gesundheitsstrukturgesetz) from 1993, already the introduction of quality management was requested [71].

The first drafts of guidelines were elaborated by Prof. Uwe Ganzer (Professor and Chair at that time of the Department of Otolaryngology of the University of Düsseldorf) and Prof. Wolfgang Arnold (Professor and Chair at that time of the Department of Otolaryngology of the Technical University of Munich) and in 1994, in the so-called Delphi procedure, the drafts were sent to leading physicians of hospitals as well as ENT specialists in private practices for review.

The drafts of the guidelines were based on the following pattern:

- Definition of the disease
- Imperatively required examinations
- Examinations required in individual cases
- Unnecessary examinations
- Therapy (conservative, surgical)
- Inpatient/outpatient treatment

Thus it is obvious that at that time also economic aspects were taken into consideration with regard to interactions with cost bearers. 

Beside the text of the guidelines, also algorithms and flow charts were developed for selected diseases. 

**Figure 1:**
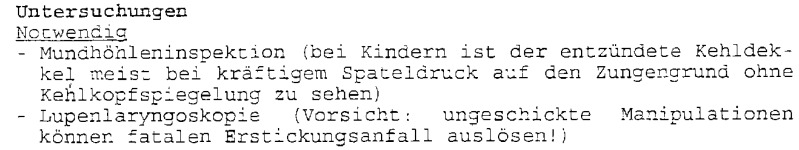
Example of an – intentional – “bug” in the first draft of the guideline on acute epiglottitis (source: author’s own archive) Translation: Examination: Necessary: – inspection of the oral cavity (in children the inflammed epiglottis may be seen by applying strong pressure onto the base of the tongue without laryngoscopy); – indirect laryngoscopy (caution: awkwardly manipulations may cause fatal seizures of asphyxiation)

Cunningly, the authors included apparent errors in the drafts of the guidelines in order to recognize if the reviewers went through the texts carefully or accepted them without reading them (see Figure 1 (Fig. 1)).

The consensus achieved by the repeated Delphi procedure was based on a three-part publication in the journal *Laryngo-Rhino-Otologie* [25], [26], [27] as well as the second edition of the Checklist of Otorhinolaryngology issued in 1997 by Arnold and Ganzer [9]. This re-elaboration led to a change from the mere character of manual of the first edition of 1990 [10].

Regarding the “analog”, i.e. paper publications of the guidelines, it has to be taken into account – and here especially the generation Y is addressed – that the internet was still in its infancy at that time and data exchange was mostly limited to FTP protocols via slow telephone connections. Web 2.0 with multifunctional browsers and uncomplicated up- and download via wideband lines was developed several years afterwards. Further, it must be remembered that online access to medical databases (e.g. Medline) was very expensive and limited to institutions. Mobile data access was not possible (it must be noted that the Apple iPhone was introduced in 2007). 

So it is quite easy to understand that the first guidelines of 1995–1996 were not based on an evaluation of evidence of scientific literature but rather on the summary of consented expert knowledge (“tradition-based”). 

Later, guidelines of this level were called S1 guidelines, disrespectfully even the acronym of GOBSAT was created (good old boys sitting around a table). 

Just to avoid misunderstandings: The development of the first guidelines of the German ENT Society was really pioneer work that had to overcome many difficulties (lack of understanding, attitude of denial, from a current point of view limited technological options etc.). The contributing parties at that time deserve the utmost respect.

Looking more deeply into the matter, it is not even true that the first German guidelines were published in 1995. 

In fact, in 1991 the journal *HNO-Informationen* published a series of articles entitled “ENT base lines – guides for Oto-Rhino-Laryngology” [48]. Apparently, this title was meant to avoid licensing disputes because the title of guidelines began prevailing as official product of the AWMF. However, it remains unclear what “base line” in this context means. 

In the following years, some authors were not hindered to call publications as guidelines that were not AWMF-verified and they even published them. Also in the Anglo-American countries publications are entitled as “guidelines” without that country-specific requirements of guideline development have been observed. 

In the following paragraphs, guidelines are defined as publications that were created according to the requirements of the AWMF, authorized and published by this association. Other German-language publications will not be cited or considered as guidelines.

The development (and application) of guidelines is a key element of the so-called evidence-based medicine. The term was introduced in 1990 by Gordon Guyatt and David Sackett of the Department of Clinical Epidemiology and Biostatistics of the McMaster University of Hamilton/Ontario [24]. The German word of “Evidenz” is misleading because the English term of “evidence” does not have the same implications as “Evidenz” [69]: evidence means “Beweis” (proof), but “Evidenz” in German may be translated with “obviousness”. However the more correct term of “nachweisorientierte Medizin” (medicine based on proven facts, evidence-based medicine) could not be established. Baethge [11] used the term of patient-oriented science with the same meaning.

In Germany, the first discussion of evidence-based medicine is associated with David Klemperer who in 1995 wrote a chapter entitled “Quality and quality control in medicine” for a textbook [36]. The main issue of evidence-based medicine is explained in this chapter: “Up to now, accepted basics of medical action and medical competence were unsystematic observations, understanding of the pathophysiology (mechanisms of disease), clinical experience, and the resulting clinical instinct or intuition. Those basics are certainly necessary, but insufficient.” (Author’s translation) 

Thus the recommendations of evidence-based medicine are based especially on randomized controlled trials [23], [29], [39], [44]. 

The application of evidence-based medicine is an integral part of quality management in medicine. 

In 2014, Nothacker et al. [52] summarized the first 20 years of work on guidelines as follows: Nowadays it can be stated that the work on guidelines has caused a cultural development in the sense of passing from recommendations of individual experts to systematically developed decision-making tools with consensus about relevant healthcare issues. Guidelines are adopted in the German Healthcare System and represent the knowledge base of numerous quality initiatives. The next aim and at the same time challenge is the establishment of a viable, theory-based concept for the implementation and evaluation of guidelines in Germany based on previous approaches. However, this shows the need of research funding. 

The mentioned publication describes the history of the development of guidelines in Germany in particular from an administrative point of view.

## 3 Changes in scientific publication: from printed documents to digital contents

After the first steps of guideline development, the system became more and more professional and regulated [6], [7], [8], [38], [40], [50], [5], [63], [66]. It was the aim to pass from consented chapters in manuals, which were still nothing else than tradition- or eminence-based publications, to guidelines corresponding to the current stage of knowledge and science according to the requirements of evidence-based medicine. 

An important precondition was the increasing expansion of digital services. 

Before the era of the digital age, relevant publications had to be found by uncomfortable searching through card boxes of libraries unless the own department had the journal as print version. 

Articles that were not available at the local library had to be ordered which was time-consuming. Often the photocopies were low-quality or even incomplete. 

At that time, one key service was provided by the journal entitled *Zentralblatt Hals-Nasen-Ohrenheilkunde, Plastische Chirurgie an Kopf und Hals* where abstracts from relevant journals were published classified according to topics. The publication of this journals was interrupted in 1996 with volume 148. 

It is easy to understand that under the former, pre-digital circumstances an extensive or even complete assessment of the literature to create guidelines was impossible. Apart from those difficulties, there were no criteria on systematic evaluation of scientific publications. 

In the further course of the time, the development of the internet allowed access also to international journals in addition to the already known journals that were often subscribed at university hospitals (e.g. Otolaryngology – Head & Neck Surgery; The Laryngoscope; American Journal of Otolaryngology etc.). The public access to the database “Pubmed” free of charge (https://www.ncbi.nlm.nih.gov/pubmed) played a major role in this context. At the beginning, only reference lists could be created, the according articles still had to be found in the own library or ordered via interlibrary loan services. 

As things developed, more and more links from Pubmed to online publications were provided so that it became possible to access complete articles with just a few clicks. Unfortunately, the expansion of the online access was associated with partly exorbitant increases in subscription prices of the journals, especially for institutional subscribers. As a consequence, independent university libraries founded associations. 

Even if today many publications may be accessed online, there are still some journals of which the articles must be ordered. A key role in this context plays the service of Subito providing documents from libraries. 

The classical print volumes of journals were completed during the last years by more and more journals that publish their articles exclusively online. Some/many of those journals request – sometimes high – fees for publication of contributions whereas the access for the user is free of charge (open access concept). The peer review process, however, is not always transparent and sometimes it is even completely omitted. Nonetheless, a significant increase of publications can be observed due to those online journals; using a neutral term, the quality of the contributions can be called “heterogeneous”. 

Graham et al. [28] stated that the number of randomized controlled trials was multiplied by 5 from 5,000 (1978–1985) to 25,000 (1994–2001) per year. Even if this is only a rough estimation, the tendency is obvious.

## 4 Meta-analyses

Because of the digitalization of scientific literature, the efforts for intensive literature researches could be clearly reduced so that the new type of publication – the meta-analyses – gained in importance. 

For this purpose, the methods of systematic analysis of scientific publications were (further) developed [35]. 

The Cochrane Collaboration is considered as most important source of high-quality meta-analyses that are called there Cochrane Systematic Reviews. While those reviews were distributed as paid CD-ROMs in former times, they are meanwhile available free of charge via library associations. The abstracts can be accessed online via http://www.cochrane.org. 

The methodology is explicitly described in an English *Cochrane Handbook for Systematic Reviews of Interventions* [32]. For the creation of a review the use of the software “Cochrane Information Management System (IMS)” is obligatory. Generally, only controlled randomized placebo-controlled trials are taken into consideration. 

The requirements to the studies that have to be included in the planned meta-analysis, however, are so high that often only very few of the identified studies are actually eligible for the meta-analysis. But it is not uncommon that also those trials are considered as being of insufficient quality. 

One hallmark of the Cochrane Collaboration is the forest plot [45] that can also be seen in the logo of the organization. Hereby, the confidence intervals of the compared trials are symbolized by parallel lines and the study power by squares of different sizes. 

The term of meta-analysis is not protected so that numerous publications in other journals are available that seem to (strictly) refer to the Cochrane criteria. Since the criteria for inclusion of trials are mostly less severe than for Cochrane, those meta-analyses are often based on higher case numbers.

## 5 Economical aspects in medical literature and guidelines

Up to now, only few medical publications deal with economic issues. In the USA, however, more and more cost-benefit analyses of certain treatment options are published. But most of the guidelines do not deal with economic and logistic aspects such as the availability of institutions for diagnostics and therapy or the approval of pharmaceutics. One example is the German S2k guideline on rhinosinusitis. In accordance with the European Positional Paper on Rhinosinusitis (EPOS 2012), the strong recommendation for application of topical corticosteroids is given for the treatment of acute rhinosinusitis. However, the problem is that none of the topical steroids available in Germany is approved for the indication of acute rhinosinusitis (apart from allergic genesis). 

A second example concerns the current S3 guideline on chronic tinnitus. The performance of tinnitus-specific cognitive behavioral therapy is strongly recommended, but the implementation of this recommendation probably leads to high capacity problems in the near future because already now qualified therapists are significantly overloaded with work.

Economic aspects will certainly play a more and more important role for the creation of future guidelines than today. The main reason will be that the individualized “personalized” therapy of malignant tumors with (highly expensive) antibodies and inhibitors (so-called biologicals) will continue increasing.

## 6 Increasing number and increasing requirements regarding clinical studies

The above-mentioned increase of scientific publications by online journals and the generally improved access to publications also lead to the fact that the individual physician is no longer able to remain always and completely informed about the current development in his discipline. While it was formerly sufficient that a scientifically working physician of a hospital knew roughly the contents of less than 10 monthly journals, a significantly higher number of journals and online publications are now available so that it is nearly impossible to be always up-to-date in the whole discipline. Hereby it must be taken into consideration that only the fact of taking note of a publication is not sufficient. Moreover the contents should be critically assessed and evaluated under the aspects of evidence-based medicine. Especially for open access journals that have no peer review system objections are justified: by paying a certain sum nearly everything may be published. 

In this context, Bastian et al. [12] analyzed the development of the number of scientific publications from 1950 to 2007. With the background of the important increase of publications, the authors come to the conclusion that an individual physician would have to read 75 new trials and 11 reviews per day in order to keep his knowledge up-to-date. 

Review articles and meta-analyses may be helpful to assess the current state of knowledge. Based on the awareness that it will be impossible for an individual physician to be up-to-date with the current knowledge of his discipline due to the multitude of publications, the concept of professional assessment of publications according to the criteria of evidence-based medicine was developed. For this purpose, requirements were established and the evaluations were mostly performed by institutions of theoretical medicine. 

The most important study results are often also reported in secondary media, generally relevant topics are even found in the lay press. Unfortunately, the editors responsible for writing such articles often do not dispose of sufficient knowledge so that often even translation errors are published. 

Another important topic in the context of trials is the publication of negative study results that is often criticized as being insufficient, e.g. output of pharmacological research. Thus the obligation should be introduced that all controlled trials are registered in a public registry and that the results are published [4], [21], [22]. The obligation to publish shall also concern already closed studies. The German Registry of Clinical Trials (Deutsches Register Klinischer Studien, https://drks-neu.uniklinik-freiburg.de/ drks_web/) fulfils the criteria for such a study registry and is acknowledged as WHO primary registry. In September 2015, the key word of ENT resulted in 40 registered trials. 

Because of the increasing observation of the principles of evidence-based medicine, the quality of clinical studies has clearly improved during the last years. In addition, high-quality journals meanwhile set highest standards for the acceptance of publications (e.g. positive ethics committee vote, registration of the study, declaration of conflicts of interest).

## 7 Guidelines – are they really appropriate?

The discussion about the usefulness of guidelines dates back to the first guidelines. While on the one hand clear instructions are requested according to the criteria of evidence-based medicine, others consider their therapeutic freedom restricted in an inappropriate manner. 

In 1997, Schneider [61] reported about an international symposium in Prien/Chiemsee, Germany, on the topic of “Classification, standards, and guidelines in otolaryngology, head and neck surgery”. He cited one of the lecturers as follows: “The demands of politicians to define medical treatment standards have to be refused because of several reasons. First, this demand is based on erroneous preconditions: in contrast to quality standards in industrial production of life- and soulless products, treatment standards in diagnostics and therapy of individual patients are unrealistic. In medicine, there is no standard patient with standard disease that can be cured by a standard physician by means of a standard method. Medicine is no exact science but characterized by the biological variability of patients and its unpredictable nature. 

Furthermore, the definition of standards cannot encompass all probabilities and constellations so that treatment standards are always incomplete and erroneous. Since they are binding, medical colleagues who do not act according to those standards would violate the rules of medical practice. Already the legal consequences that might results justify the requirement to elaborate medical standards with highest diligence and care. The introduction of new methods in diagnostics and therapy of the patients could also be impaired by defined standards so that medical progress would be impeded. The German Society of Oto-Rhino-Laryngology, Head and Neck Surgery should thus limit its statements on directives or recommendations.” 

The author of this present contribution would like to point out that those statements were a general historical misjudgment. Standards and guidelines are meanwhile an established part of scientific medicine.

The current definition of guidelines is as follows (based on the AWMF guidelines that is valid at a certain time): systematically developed descriptions and recommendations with the objective to support physicians and patients in the decision-making process of finding adequate measures of medical care (prevention, diagnostics, therapy, and follow-up) under specific medical circumstances.

In 2011, the Institute of Medicine [28] issued the following new definition of guidelines: “Clinical Practice Guidelines are statements that include recommendations intended to optimize patient care. They are informed by a systematic review of evidence and an assessment of the benefits and harms of alternative care options.” 

The clinical practice guideline on cerumen [58] may serve as an example for the fact that guidelines are also elaborated on topics where seemingly no need for regulation can be seen – but at least the document has 15 authors and 21 pages. The same may be true for the below-mentioned trial on the application of dexamethasone and antibiotics in the context of tonsillectomy [55].

### 7.1 Stages in the development of guidelines

Since the beginnings of guidelines, their development was significantly professionalized and institutionalized. The AWMF as umbrella organization of medical scientific societies in Germany has developed and further improved the most important directives [6], [7], [8], [5]. 

Guidelines are classified into three stages or levels of development, i.e. S1, S2, and S3, while stage S2 is subdivided into S2e and S2k since 2004 (Table 1 (Tab. 1)). The determination of the stage has to be performed during the registration process of the guideline. For guidelines of stage S2 and higher is required to describe the applied methods in a guideline report because of better transparency. This guideline report is published as a separate document. 

Regarding S2k and S3 guidelines, formalized consensus procedures have to be passed [64]. Those formalized consensus procedures are [41]:

- Nominal group process
- Delphi technique
- Consensus conference

Those 3 procedures are applied in particular depending on the group sizes.

The AWMF issued the principle to no longer register S1 guidelines in the future. S1 guidelines that would have to be actualized should preferably be upgraded. Unfortunately, this recommendation is currently neglected in some cases. 

Guidelines of the stages S2e and S3 require systematic literature analyses that has to be performed based on defined rules. This is the largest cost factor in the context of the development of a guideline. 

Guidelines with oncological topics may be funded by the German Cancer Society as oncological guidelines (onkologische Leitlinien, OL) [53]. The same is true for national healthcare guidelines (nationale Versorgungsleitlinien, NVL) that are financed with public resources. 

In some cases, short interdisciplinary guidelines are published as booklet by the German Cancer Society [19], [20]. 

The numbers of currently applicable guidelines of the German Society of Oto-Rhino-Laryngology, Head and Neck Surgery are listed below according to the stages:

- S1: 8
- S2: 4 (guidelines have been developed before the subdivision into S2e and S2k was made)
- S2k: 7
- S2e: 0
- S3: 1

The only S3 guideline that was elaborated by the German Society of Oto-Rhino-Laryngology, Head and Neck Surgery is the guideline on chronic tinnitus. 

Another relevant S3 guideline deals with the oncological guideline on carcinomas of the oral cavity. The observation of single recommendations of this guideline is verified during the certification process of head and neck tumor centers by the German Cancer Society. 

Tables 2 (Tab. 2) and 3 (Tab. 3) show the current guidelines of the German ENT Society (Table 2 (Tab. 2)) as well as guidelines to which the German ENT Society contributed (Table 3 (Tab. 3)). For some guidelines, the validity date had expired at the time of printing of this present article.

### 7.2 Conflicts of interest

In the last years, the focus was more and more placed on the conflicts of interest that had to be taken into consideration by the authors of guidelines [43], [46]. Since it seems to be unrealistic to allow only contributions to guidelines made by people without conflicts of interest, the disclosure of potential conflicts of interest was required by the AWMF. This is either made within the text of the guideline or as a separate document. Authors with such conflicts of interest are encouraged to abstain from respective voting. The discussion of the influence of third parties on guidelines has already reached the lay press. Spiegel online published an article written by N. Kuhrt entitled “Mediziner warnen: Pharmaindustrie soll Leitlinien beeinflusst haben” (Physicians warn: pharmaceutical industry is suspected to influence guidelines). As an example, the author mentions the early inclusion of new pharmaceutics in guidelines without that they had been applied in clinical routine for a longer period.

### 7.3 Adaptation of international guidelines

Of course, guidelines are also developed in other countries. In an editorial or rather comment on a special issue of *Otolaryngology, Head and Neck Surgery*, Rosenfeld [60] discussed the first Clinical Practice Guideline of the AAO-HNS on the one hand as milestone, on the other hand, however, as a beginning or starting point. This guideline dealt with acute otitis externa.

The international platform for guidelines is the Guideline International Network [56], G-I-N.net. On the website, among others the International Guideline Library with currently 6,182 guidelines from 75 countries is found (as of September 30, 2015). 

Regarding this international guideline registry, the question must be asked why every country or every national society undertakes the efforts to establish their own guidelines. At a first superficial glance, it seems to be possible to safe many human and financial resources if already existing guidelines were translated and adapted. However, there is the problem that country-specific requirements in terms of methodology (e.g. literature research, assessment of evidence, formal requirements to the consensus process, conflicts of interest) could not be considered. Even country-specific particularities such as availability and approval stage of pharmaceutics limit the use of international guidelines so that there will probably be no internationally applicable guidelines. National requirements for the development of guidelines additionally impede such projects (see for example the American manual on the development of guidelines [59]).

### 7.4 Bindingness of guidelines

Current guidelines reflect the current state of knowledge (results of controlled clinical trials and expert knowledge) about effective and appropriate healthcare at the time of printing. Regarding the fact that scientific knowledge and techniques progress, revisions, renewals, and corrections have to be performed periodically. The recommendations of guidelines cannot be applied adequately under all circumstances. The decision if certain recommendations has to be followed, must be made by the physician taking into account the circumstances and conditions of the individual patient and available resources (author’s translation of [72]). 

Because of the regularly expected need for actualization, guidelines have an “expiry date”, after that date the guideline is no longer valid. The responsible society can apply for extension of the validity to the AWMF if there is no obvious need for revision. Vice versa, a society may modify or complete a guideline during its validity period if new findings indicate this.

**Guidelines give recommendations for standard situations and are expected to provide corridors of action in standard situations.**

This makes them different to directives on the one side and recommendations on the other [49]. 

Directives have a binding character if they were issued by a legally, profession-related, quality-related, or statute-related legitimate institution with respective legislative competence. Their non-adherence may be punished. Examples for directives are the directive on the collection of blood or blood components and on the application of blood products (hemotherapy) issued by the German Medical Association (Bundesärztekammer, BÄK) (not to be confused with the horizontal guidelines on therapy with blood components and plasma derivates), the directives on pharmaceutics, the directives on maternity, and the directives on patient transport [72]. 

Recommendations on the other hand are non-binding statements that are given either by single persons (e.g. consiliary physicians) or a group (e.g. medical society, work group, conference) for a specific single case or in general. The non-adherence of recommendations does not have legal consequences. 

The recommendations to assess noise-induced hearing loss (“Königsteiner Empfehlung”) is called “recommendation” and at first sight it does not seem to be binding. In fact, they are actually considered as anticipated expert report. This means that an assessment of hearing loss based on the requirements of this recommendation can be legally valid without consulting a medical specialist in person. Especially for administrative procedures with professional associations and insurances, this aspect is important because noise-induced hearing loss without further particularities can thus be assessed and confirmed by non-medical staff.

Guidelines are not legally binding, there is no law and no regulation that penalizes the non-adherence to a guideline. Since guidelines on the other hand represent mainly the current state of the art of medical science in the respective discipline (requisite care, adherence to rules of medical practice), i.e. describe current medical standard, guidelines are considered as important source of knowledge for actions that can be demanded from a physician according to his professional standard. To what measure treatment that is not in conformity to guidelines has to be considered as violation of the care standards, must be verified in the individual case [72]. According to the same reference, however, a certain change in legislation is visible that statements from guidelines can be taken as measure of standard from a liability-based point of view. 

This change is also revealed by 2 decisions of the Federal Supreme Court:

A resolution of 6^th^ civil senate dated March 28, 2008 about a complaint stated that the guidelines of medical committees or associations cannot be put on the same level with required medical standard for assessment of treatment errors (in contrast to directives of the Federal Committees of physicians and health insurances). They cannot replace expert reports and not be blindly accepted as measure of standard. Finally, the definition of the standard is based on the assessing of the judge whose decisions can only be verified regarding legal and procedural errors, in particular regarding infringements of rules of logic and common experience, if the court misunderstood or did not exhaustively assess the concept of medical standard or the submitted facts (author’s translation).

The same senate (with a new president) decided on February 7, 2011, regarding a non-admission complaint that according to the guidelines, perioperative antibiotic prophylaxis had to be performed for the implantation of osteosynthetic material in order to avoid postoperative wound infection. 

The court of appeal would have to have called a legal expert to complete this presentation regarding the question if such guidelines had already been published at the time of treatment and if they corresponded to medical standard at the time of treatment. In such a case, the matter would have had to be clarified with the support of the expert if the non-adherence to the guidelines was a severe treatment error that might have led to a reversal of the burden of proof. In contrast to the opinion of the court of appeal, the presentation of the plaintiff was sufficient because the consulted expert referred to the publication of the AWMF and this was sufficient as source without further comment since the AWMF published the guidelines of the specific societies in “AWMF online”, which is publicly accessible by everyone. (…) Even if the guidelines do not have constitutive significance, the court of appeal should not have ignored the plaintiff’s description without asking a legal expert (author’s translation).

So a treating physician is always recommended to document a reasonable justification in cases of intended violation against valid recommendations of guidelines. This is especially true for “high-quality” S2 and S3 guidelines. In cases of possible later legal dispute, this measure allows providing a proof that the guideline was not violated because of lacking knowledge, but that is was justified in this individual case. Vice versa, a deviation of guidelines can only be considered as violation of due medical diligence or treatment error if the guideline represents the current standard and in the single case a deviation of this standard is not justified. However, those aspects have to be regularly clarified by expert reports. 

According to prevailing opinion, guidelines are no anticipated expert reports because they regularly reveal corridors for action, i.e. treatment alternatives, and are not legally binding directives. This means that legal disputes always have to assess the individual case – by asking for medical expert reports [73].

**It is strongly recommended to justify planned or performed deviations of valid guidelines in a comprehensible way. A violation that is not sufficiently justified, represents a liability risk, especially if the guideline has a higher development stage (higher than S1).**

Of course, a physician of a specific discipline can be expected to know about the existence and the contents of the guidelines concerning his specialty. This is already justified by the obligation to pursue regularly the continuous medical education programs [16]. It must be mentioned again that this also applies for guidelines that have not been developed by the own society but were consented by it. Those guidelines are listed in Table 3 (Tab. 3). 

It is a very particular case when 2 guidelines on the same or similar topic are available from 2 different societies. Even if the central registration of guidelines at the AWMF should avoid such a doubling, there is an S3 guideline on rhinosinusitis of the German Society of General and Family Medicine (Deutsche Gesellschaft für Allgemeinmedizin und Familienmedizin, DEGAM) (053-012) and an S2k guideline of the German Society of Oto-Rhino-Laryngology, Head and Neck Surgery (017-049). Meanwhile, the validity of the DEGAM guideline has expired. But nonetheless it is problematic if 2 parallel guidelines exist on the same disease; in analogy to the specialist standard, a guideline is only valid or liable for the according discipline. The general practitioner is bound to the DEGAM guideline, the otolaryngologist to the guideline of the ENT Society.

### 7.5 Grades of recommendation of guidelines

As mentioned above, the recommendations (this term can be misunderstood from a legal point of view, it seems to be better to use “statements”) given in guidelines are classified into different grades that are based on the level of the existing evidence. 

The following statements in guidelines are established (Table 4 (Tab. 4)):

- Must
- Should
- Can
- Should not
- Must not

In general, these statements follow the elaborated level of evidence or grade of recommendation (A, B, 0, see Table 1 (Tab. 1)). 

Must-statements generally have such a high grade of recommendation that their observation seems to be obligatory. If particularities occur in single cases justifying a deviation, it should be explicitly documented. On this premise, deviations can of course be justified. 

The grade of recommendation may change due to new evidence or re-assessment of study data during revision of a guideline. 

A good example in this context is the guideline on sudden hearing loss. 

In the previous version of the guideline, the intravenous administration of 250 mg prednisolone on 3 subsequent days was still highly recommended (must-recommendation). A physician who did not follow this statement was well-advised to justify a deviation of this procedure (e.g. oral application of lower doses, primarily intratympanic administration). Due to a re-assessment of the literature, the grade of recommendation was modified to “should” in the current version of the guideline. 

In July 2015, the therapy of sudden hearing loss was the topic of a message of the Medical Service of the Association of Health Insurances entitled “Kortison hilft nicht beim Hörsturz” (Cortisone is ineffective for therapy of sudden hearing loss; https://www.mds-ev.de/presse/pressemitteilungen/2015/2015-07-09.html). The key sentence of this publication was that there was no hint to the benefit of systemic application of glucocorticoids for therapy of sudden hearing loss based on congruent statements published in significant review articles. 

So there is a clear discrepancy between valid recommendations of guidelines and this statement of the Association of Health Insurances that may lead to a great uncertainty of the patients. This message will probably fan again the discussion of therapy of sudden hearing loss, also because the German Society of Oto-Rhino-Laryngology, Head and Neck Surgery published a respective reply together with the Professional Association of Otolaryngologists. It is also mentioned here that the author of this present article has a general problem with considering the intravenous injection/infusion of prednisolone as individual healthcare service and not as treatment covered by statutory health insurances, also because the – imprecise – approval for such pharmaceutic products does not explicitly include the indication of sudden hearing loss, but it does not exclude it either. 

Guidelines from other countries may have a significance especially if a concrete guideline issued by the AWMF is missing or if the validity date has expired (see also chapter 7.3). 

In some cases, German as well as European guidelines are available, the best example is EPOS 2012 and the AWMF guideline on rhinosinusitis (i.e. the guideline of the German ENT Society and not the DEGAM guideline). Both guidelines are not always congruent. In this context the general rule may be applied that in cases of non-accordance with the European guideline the national guideline is superior. If the treating physician recognizes a contradiction, he is well-advised to document why he follows which guideline. 

Despite their often very high quality, guidelines from outside Europe are regularly considered as not binding; in other words, no physician can be accused for not including the US American or Peruvian guideline into his therapeutic concept. 

Another question is if the treating physician must be expected to know the respective guidelines. This question was already answered with yes (see above). 

The – cost and time consuming – development of guidelines must lead to the claim that physicians of the respective discipline have to know the contents of the specific guidelines. 

Nowadays, guidelines may be easily retrieved via different ways (http://www.awmf.org/; http://www.leitlinien.de/; https://www.coliquio.de/; and other online platforms). Since hospitals and practices should dispose of an access to the world wide web, the argument of lacking internet access seems to be far from reality (and has never been used in questions regarding expert reports to the author of this article). 

The obligation to know interdisciplinary guidelines is extended also to those guidelines that were established by other responsible societies but involving the own society. Those guidelines can be easily identified on the AWMF homepage (see also Table 3 (Tab. 3)). 

Explicitly, the following particularly relevant interdisciplinary guidelines are mentioned:

- Antibiotic prophylaxis
- Prophylaxis of venous thromboembolism
- National management guideline of asthma

On the other hand, a specialist cannot be expected to know and if necessary to apply the guidelines of other disciplines. Exceptions are seen when a specialist treats patients with diagnosis of an actually different discipline (e.g. an ENT specialist diagnoses an episode of depression in a patient suffering from tinnitus and prescribes antidepressants or an ENT specialist issues a follow-up prescription for a treatment that was initiated by a colleague from another discipline). 

Hereby, problems occur regularly especially in the context of inpatient treatment in daily routine when the treating ENT physician recommends to continue the own medication during the inpatient stay. A physician on a ward may rely generally on the correctness of a medication plan of a colleague in the sense of horizontal division of labor, but on the other hand he has to check the medication plan for apparent inaccuracies (e.g. simultaneous application of an active substance in form of several generic products, obviously erroneous dosage, combinations of interactive or counterproductive drugs, wrong application intervals). 

If in the course of inpatient treatment another pharmaceutic product is additionally prescribed, the treating physician is responsible to check the overall medication with regard to interactions (e.g. combinations of drugs that compete for the same metabolism pathway). 

## 8 Choosing wisely

Choosing wisely (http://www.choosingwisely.org/) is a campaign that was initiated in 2012 by the ABIM Foundation (Advancing Medical Professionalism to Improve Health Care) [15]. The aim of this campaign was to give recommendations for daily medical routine in an easily formulated way while especially non-indicated, counterproductive medical measures were emphasized. So the initiators mainly had medical overprovision in their focus. 

The American Academy of Otolaryngology – Head & Neck Surgery Foundation participated in this campaign by publishing 10 statements listed in Table 5 (Tab. 5). Without any doubt, also in Germany those recommendations are violated day by day.

In Germany, the discussion was started to take up this initiative and to transfer it to German circumstances. This led to establishing an ad-hoc commission involving also the AWMF entitled “Gemeinsam klug entscheiden” (making wise decisions together). 

Also the annual meeting of the German Society of Internal Medicine in 2015 had this motto [31]. 

But also criticism regarding the choosing wisely campaign was expressed. One aspect was that the methods leading to the single statements were unclear and not standardized. Also the first steps toward prioritization or economization in healthcare were expected to be recognized. Further criticism was based on the fact that mostly don’ts and no dos were formulated. 

Even if the criticism was at least partly justified, it must not be forgotten that the choosing wisely statements were developed in particular for communication with lay people and had the primary objective to avoid unnecessary medical measures. Mentioning the respective “official” statement of the society, a physician may for example explain easily why he does not initiate the desired diagnostic procedure (e.g. radiography of the paranasal sinuses) or why he does not prescribe a drug (e.g. systemic antibiotics for otitis externa) without being considered as the scrooge who is the enemy of patients and the friend of health insurances. 

The necessity of listing the negative recommendations was revealed by an investigation published by Klemperer and Dierks [37]. A survey of the population concerning the assumed performance of unnecessary services in medical practices showed that 57% expected a high frequency, 31% replied “sometimes”. The question of omitting useful services was replied with “very often” or “often” in 39% and with “sometimes” in 38% of the participants. 

Reifferscheid et al. [57] asked leading hospital physicians to what extent they recognized overprovision in their discipline. A definite “yes” was given by 24.8% of the cardiologists, 20.5% of the trauma surgeons and orthopedists, and 17.2% of the anesthesiologists as well as 11.4% from other disciplines. The study does not reveal if also ENT specialists were included.

## 9 Implementation of study results in guidelines

Today, it seems to be nearly impossible for every (ENT) physician to be always up-to-date of the current scientific knowledge. This is especially true for publications apart from the own discipline. Thus there is no other option than to benefit from prepared meta-data. Those meta-data are for example meta-analyses (e.g. Cochrane reviews), current CME articles, and guidelines. However, it is obvious that a timely delay must be calculated between the publication of original articles and the publication of meta-analyses or guidelines. 

Typically, guidelines are initiated by the respective society that is primarily responsible for the disease or symptom. In this context, the AWMF supports in particular guideline projects with interdisciplinary approach, i.e. guidelines as of development stage S2. 

A systematic assessment of the available scientific literature in the sense of evidence evaluation is only intended for S2e and S3 guidelines. 

This evaluation of the literature has to follow strict methodical requirements that are defined in the AWMF regulations [5]. They encompass the following steps:

- Definition of the search strategy
- Definition of inclusion and exclusion criteria
- Documentation of the selection
- Assessment of the methodical quality
- Creation of evidence tables
- Definition of the level of evidence

Hereby, first guidelines of other, also international, societies have to be looked up and then systematic review articles (aggregated evidence) before performing a research of the primary literature. The primary literature research may be limited to controlled trials. The exact search strategy has to be documented. Furthermore, it is recommended to use several, but at least 2 databases (e.g. Medline, Cochrane Library, Embase) because Medline does not encompass all medical publications. 

Needless to mention that the complete texts of the identified literature have to be read and assessed. The AWMF considers as desirable that 2 reviewers evaluate the literature independently (and come to the – hopefully – same conclusions). 

The evidence tables have the following scheme:

- Reference
- Type of study
- Participants (number and characteristics), total and per study arm
- Drop-out rate
- Intervention
- Control
- Target value(s)
- Main result
- Annotations
- Conclusion of the authors of the trial
- Conclusion of the reviewers

The levels of evidence are defined based on a previously fixed scheme. Known classifications are:

- Oxford classification (Table 6 (Tab. 6))
- SIGN classification (Table 7 (Tab. 7))
- GRADE (Table 8 (Tab. 8) [62])

Based on the determined level of evidence, the evidence grade (A, B, or 0) is then defined and the recommendation is formulated (must, should, can etc.). 

In summary, it can be stated that the study results find their way into a guideline via systematic literature analysis that is only required in the context of S2e and S3 guidelines. 

In theory on the other hand, S1 and S2k guidelines may do without any literature citations. But regularly, also S1 and S2k guidelines use literature references, however, it must be clear that methodical flaws may occur in the context of the selection and evaluation of the literature.

## 10 Implementation of medical guidelines in daily routine

It can be easily understood that the high staff-related, timely, and financial efforts of establishing a guideline are only justified if the (regular) adherence to guidelines leads to a measurable benefit for the patients [65]. 

A classic example for the proof of such a benefit is the investigation of Varga et al. [70] from 2010. The survival rates of 1,778 female patients with early breast cancer were calculated in dependence on the adherence or non-adherence to the German S2 guideline on breast cancer. In this context, a significant benefit in favor of adherence to the guideline was observed in terms of the recurrence-free survival as well as the overall survival (overall survival: p<0.001; HR 2.92 [2.01; 4.23]). 

This study shows a significantly improved quality of the outcome by adherence to guidelines. 

Comparable investigations from Germany in the field of otolaryngology still do not exist, they are urgently needed in the future.

Many investigations on adherence to guidelines (publications with ENT-specific topics are presented below) only focus on the process quality, i.e. the question if the recommendations of the guidelines are implemented at all (and not if the implementation of the recommendations has an impact on the treatment outcome, which is actually the more important question).

**Figure 2:**
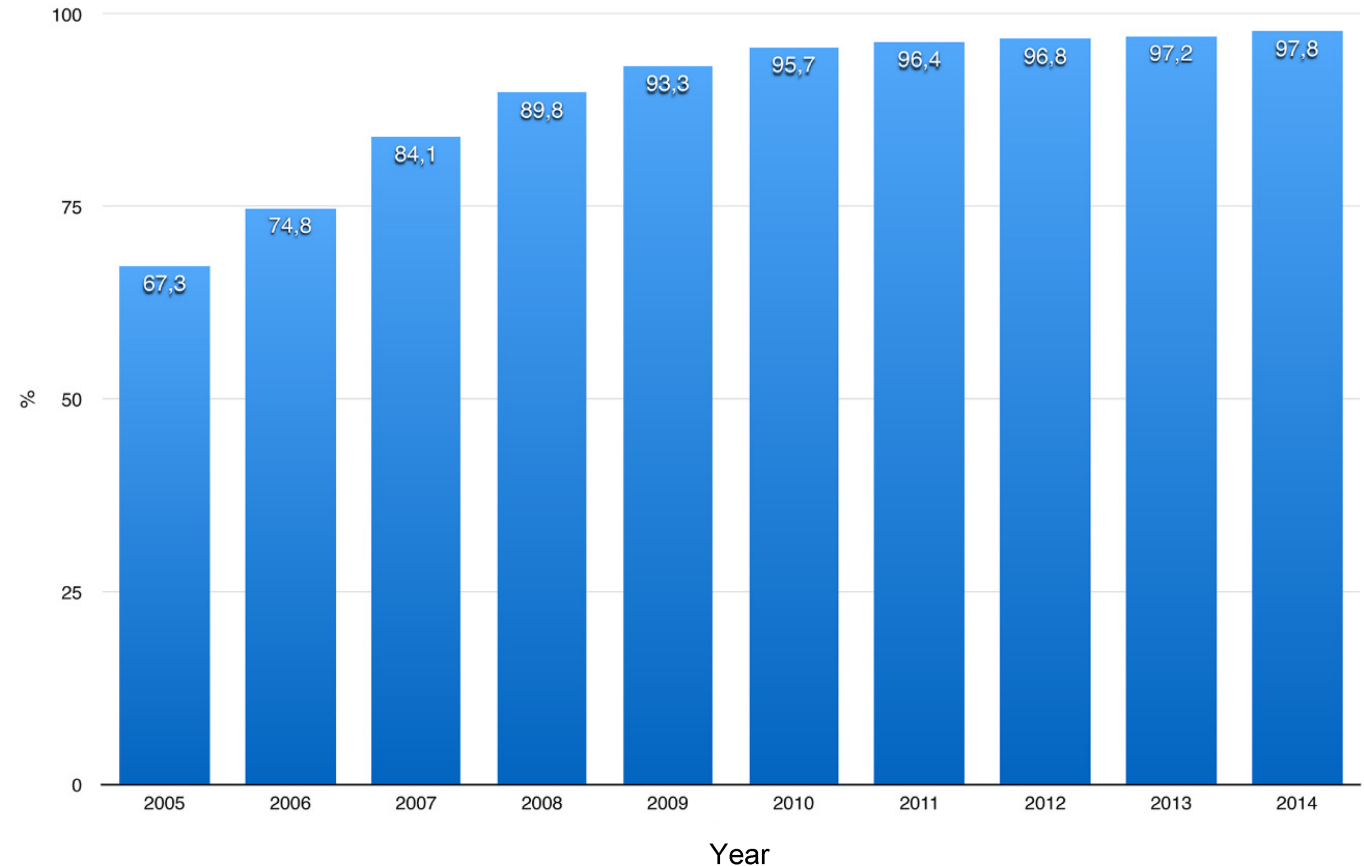
Definition of the quality indicator of “blood gas analysis/pulse oximetry within 8 hours after hospitalization” in the context of community acquired pneumonia between 2005 and 2014 (source: Aqua Quality Reports 2005–2014)

One example is found in the context of the guideline on community-acquired pneumonia. This guideline demands first blood gas analysis or pulse oximetry within 8 hours after hospital admission. It is a quality indicator that is nation-wide collected for quality reports by the Aqua Institute on behalf of the Federal Joint Committee. The target value is >95%. Figure 2 (Fig. 2) shows the development of this parameter from 2005 to 2014. Thus, the objective of the guideline was continuously achieved since 2010. If this development led to a reduction of the mortality of community-acquired pneumonia, still remains open.

Aarts et al. [2] investigated the adherence to guidelines of otolaryngologists in the Netherlands (N=440, response rate: 187/440=42.5%) by means of questionnaires. Hereby, 70% of the responders stated that they observed guidelines in daily routine, 61% did so every day, 20% 2–3 times per week, and 19% once per week or more rarely. 62% considered guidelines as helpful support. The application rate of guidelines was significantly higher in academics than in generalists (p<0.05). Especially oncological guidelines were more popular for physicians in university hospitals than in private practices.

The AWMF suggests the following theory regarding the implementation or non-implementation of guidelines [51]:

- Cognitive theory: the lack of knowledge inhibits the implementation
- Behavioral theory: missing incentives, feedback, and external stimuli
- Social theory: missing social pressure by superiors
- Selling theory: unattractive marketing of knowledge and action
- Organizational theory: weakness of the system

In practice, a mixture of those 5 theories seems to be realistic for the non-implementation of guidelines.

According to Leon Festinger, the cognitive dissonance is an adherence to previous behavior despite obvious evidence for a modification of this behavior. This cognitive dissonance is one reason for not implementing or at least a delay in implementing new knowledge in daily practice. Examples are easily found in daily routine, e.g. the application of dye, hydrogen peroxide and iodine containing substances for wound treatment, prolonged administration of antibiotics in situations of prophylaxis, use of topical antibiotics (except for otitis externa), initial prescription of systemic antibiotics for uncomplicated otitis media and rhinosinusitis etc.

Another reason for the missing adherence to guidelines may be that the guidelines do not meet the needs of practice, i.e. that they cannot be applied in daily routine. One must bear in mind that evidence is generated based on study results and that patients involved in trials are different from “normal” patients because of respective inclusion and exclusion criteria and other particularities. Anyhow, guidelines are only applicable in standard situations. So the quality of a guideline can also be measured to what extent it takes into account conditions of real daily practice. 

Furthermore, it must be taken into consideration that the implementation of recommendations of guidelines may fail due to the patients’ preferences (e.g. the wish of “natural” medication, refusal of guideline-conform surgery, choice of treatment near the home town without a specialized center). 

The implementation of current guidelines in clinical routine can also be impeded by economic aspects.

Current German guidelines regularly include study results that have been gained outside of Germany. As a consequence, possibly different approval status of the applied medication must be expected (see above: the administration of topical steroids in acute rhinosinusitis). 

The application of pharmaceutics without the specifically approved indication is possible in the context of medical therapeutic freedom. It is then called off-label use. However, it must be clear that it is not possible to make liability claims against the pharmaceutic company in cases of off-label use. Unless off-label use is planned, the patient has to be thoroughly informed and agree to the treatment. Of course, all costs related with the off-label use have to be borne by the patient himself. 

In its decision of December 6, 2005, the so-called “Nikolausbeschluss”, the Federal Constitutional Court defined criteria when statutory health insurances have to reimburse the expenses of off-label use:

It is not in accordance with the fundamental social rights that members of public insurance schemes who suffer from a life-threatening or regularly lethal disease for which treatment of acknowledged medical standard is not available, are excluded from treatment methods that might be helpful in terms of healing or at least noticeable improvement of the course.

Those 3 conditions all have to be fulfilled. The Federal Social Court of Germany generally requires for the criterion “treatment success” double-blind, randomized, placebo-controlled or at least equivalent trials; case reports are not sufficient. In cases of unresearched, life-threatening, regularly lethal, catastrophic, or rare diseases, the criteria are less severe, hereby hints for possible, noticeably positive effects suffice. 

The statements above only refer to outpatient treatment by panel physicians. This means that only treatment procedures may be reimbursed by the statutory health insurances that are explicitly labelled as service covered by the health insurances (authorization right). New diagnostic and therapeutic methods have to be checked and verified if they are useful, necessary, and economic.

In the inpatient sector, however, the principle of reservation of prohibition was applied, i.e. all treatment procedures could be applied unless they were not explicitly excluded from the care of panel physicians by the Federal Joint Committee. The Federal Social Court of Germany has questioned the concept of the prohibition right in the inpatient sector by several decisions by considering § 137c SGB V as procedural rule and not as general acceptance of the reservation of prohibition. If this view had been generally acknowledged, it would have had an enormous impact on study projects in an inpatient setting. The legislator, however, decided to maintain the reservation of prohibition by completing § 137c SGB V accordingly (see GKV Versorgungsstärkungsgesetz dated July 16, 2015). It defines that diagnostic and treatment methods for which the Federal Joint Committee has not made a final decision may be applied in hospital care if they have the potential of a necessary treatment alternative and their application is performed according to the medical state of the art, i.e. it is medically indicated and needed. This applies for methods that are not yet approved as well as for methods that are still in the stage of approval. 

Thus legal certainty has been re-established.

In order to improve the adherence to guidelines, the AWMF suggests the following measures considered as being “generally effective”:

- Audit on site
- Manual or electronic reminders
- Interactive education
- Combined strategies

The following interventions are considered as “sometimes effective”:

- Check and feedback
- Involvement of local opinion leaders
- Local consensus processes
- Involvement of patients

“Rarely effective” are the following interventions:

- Distribution of guidelines in written form
- Passive education (e.g. lectures)

As a consequence, also this present article will not provide an important contribution to improve the adherence to guidelines …

Regarding the adherence to valid guidelines in clinical routine no investigations on German otolaryngologists are currently available, as mentioned above. So some respective publications from other countries will be presented.

Al-Hussaini et al. [3] investigated the influence of national guidelines on the tonsillectomy rate and the incidence of acute tonsillitis episodes in Great Britain. In 1999, the SIGN guideline 34 (Scotish Intercollegiate Guidelines Network) was introduced that limited the indications of tonsillectomy. In a retrospective study, 699,898 tonsillectomies from 1999 to 2010 and admissions to hospitals because of tonsillitis or peritonsillar abscess were evaluated. Linear regression revealed for 2 of 3 countries a significant decrease of tonsillectomy (England: p=0.005; Wales: p=0.03; Scotland: p=0.24). During the same time, however, in all 3 countries, the number of hospital admissions because of acute tonsillitis significantly increased. In England and Wales, also the incidence of peritonsillar abscesses increased. The authors drew the conclusion that the criteria for the indication of tonsillectomy established in the guideline were possibly too strict.

A very recent study of Padia et al. from 2015 [55] deals with the adherence to the AAO-HNS guideline on tonsillectomy in children with regard to the recommendations of intraoperative single shot administration of dexamethasone and against routine antibiosis. Both are “strong recommendations”. From 2008 to 2014, 15,950 cases (children between the ages of 1 and 18 years) were analyzed that underwent adenotonsillectomy in 19 hospitals performed by 74 surgeons. Before introduction of the guideline in January 2011, 7,432 children underwent surgery, afterwards 8,518. Dexamethasone was administered in 98.4% before introduction of the guideline, afterwards in 98.9%. Antibiotics were perioperatively applied in 16.1% (before the introduction of the guideline) and in 13.8% (afterwards). While before introduction of the guideline 36% of the surgeons applied antibiotics routinely, still 26% did so afterwards. 

This publication depicts clearly that there was actually no need for regulation concerning the administration of dexamethasone. However, also with regard to the (not recommended) antibiosis, the guideline could not provide significant impulses because still a quarter of the surgeons turned out to be resistant to guideline adherence.

A study performed by Silva et al. [67] from Manchester, focused on the adherence and correct documentation of the indication of tonsillectomy. Based on the SIGN guidelines applicable in England, tonsillectomy is indicated when severely impairing sore throat episodes are documented at least 5 times during at least one year. A prospective data collection revealed that only in 2 of 17 tonsillectomy patients the documentation about the indication process was in conformity to the guidelines. After intervention in form of a reminder letter, correct documentation was found in 85 of 100 cases.

Aarts et al. [1] observed the adherence to guidelines in 217 patients with cancer of the oral cavity of stages II and III who had been treated at the University Hospital of Utrecht from 1991 to 2001. The guideline to be applied was the regional guideline of the Integral Cancer Centre Middle Netherlands (ICCMN). Adherence was found in 55%. In the context of missing adherence to the guideline, overtreatment was found in 21% and undertreatment in 71% of the cases, 8% of the patients were affected by both errors. Surprisingly, the violations of the protocol had no impact on the recurrence rates, not even the undertreatment.

A very recent investigation was performed by Hall et al. [30] from Ontario/Canada about the guideline on simultaneous radiochemotherapy from 2000. It is a retrospective evaluation of 571 cases from 9 centers from 2003 to 2004. In 55%, the respective literature was observed, while the adherence in the centers varied between 39% and 82%. Only 4 of the 9 centers applied the recommendations of the guidelines in the majority of their patients. Regarding the prognosis, no significant difference could be identified in the single centers (p=0.64).

Another study conducted by McKie et al. [47] dates back to 2008. According to the Department of Health Guideline from 2000, every patient in England with suspected head and neck malignoma should present to a specialist within 2 weeks. In this context, 10 symptoms and hints were defined. The authors assessed 1,079 consultations. Malignoma was diagnosed in 10.9% of the cases. In 71.5%, the criteria of the guideline for such an urgent consultation were actually fulfilled, the most frequent reasons for misuse of the regulations were sore throat, reflux complaints, and globus sensation. If the criteria were fulfilled, malignoma was detected in 12.8%. If not, the detection rate was only 6.2% (p<0.01). So the program had a sensitivity of 83.0% for the detection of head and neck cancer with a specificity of 30% and a positive predictive value of 12.8%.

Bhattacharyya and Kepnes [13] investigated the adherence to guidelines in acute bacterial rhinosinusitis in adults in the USA. For this purpose, the periods of 2005–2006 (7.9±0.9 million medical consultations) and 2009–2010 (10.2±1.5 million consultations) were compared; the Adult Sinusitis Clinical Practice Guideline had been introduced in 2007. The adherence to the following recommendations was assessed:

- Recommendation against radiological diagnostics (except from complications or relevant differential diagnoses): 2.3±0.7% vs. 3.5±1.4%. Because of the high standard deviations, no further analyses were performed.
- Strong recommendation for pain therapy: in this context, no significant changes were observed comparing both periods (18.9% vs. 23%, p=0.470).
- The option of observation without antibiotic treatment: 75.5% received antibiotics before introduction of the guideline, afterwards 85.7% (p=0.021).
- The recommendation to use amoxicillin in cases of antibiosis was observed in 8.1% vs. 29.4% (p=0.001).

Another publication on rhinosinusitis was also written in the USA [18]. The adherence to the recommendations of the guideline by 10 physicians was verified based on the data of 90 patients who consulted an ENT department. In 76 patients, chronic rhinosinusitis was diagnosed, in 11 patients it was acute bacterial rhinosinusitis, and in 3 patients acute viral rhinosinusitis. In cases of chronic rhinosinusitis, the 7 verified criteria were observed in 4% (differentiation between chronic rhinosinusitis and recurrent acute rhinosinusitis) to 88% (confirmation of the diagnosis), in the context of acute bacterial rhinosinusitis in 0% (differentiation between bacterial or viral genesis) to 41% (option of observation). None of the recommendations was kept in cases of viral rhinosinusitis.

Worley et al. [75] investigated 100 outpatients who presented with the suspected diagnosis of (chronic) rhinosinusitis. Only because of the clinical suspicion, 50 patients underwent computed tomography of the paranasal sinuses. The indication for computed tomography was only made in the other 50 patients when the preconditions of the “guideline” developed by the authors themselves were fulfilled. The Lund-Mackay score was calculated for the findings of computed tomography. For the first group, a score of 5.57 was revealed, for the second group, a score of 8.62 was found. This study shows that a more critical indication of computed tomography in cases of suspected chronic rhinosinusitis leads to a higher percentage of pathological findings. This fact could have been predicted. Anyhow, the criteria applied here, cannot be considered as guidelines in the sense of the definition.

The abstract of the paper of Black and Hutchings [14] from England starts with the disillusioning sentence: “It is widely accepted that the passive dissemination of national clinical guidance has little or no impact on practice.” This investigation deals with an “Effective Health Care Bulletin” on surgical therapy of mucotympanon (glue ear) from 1992. From 1975–1985, the frequency of this intervention increased from 53.2 (related to 10,000 children younger than 10 years) to 129.5 and from 1986 to 1992 it slightly decreased from 131.8 to 121.8. After publication of the bulletin, the rate decreased until 1998 from 119.9 to 68.4. Thus, an effect due to the guideline was observed even if the authors recognized a more and more critical attitude of the patients because of publications in the lay press.

Keeley [34] wrote a letter-to-the-editor concerning this article questioning the relevance of the guideline or bulletin for the described effects [17].

Another article to be presented here is from Taiwan and deals with the guidelines on acute otitis media in children. The authors investigated the adherence to guidelines regarding the treatment of children between 2 and 12 months of age who were treated between 2005 and 2008 in an ENT department in Taipei. The guideline was established in 2004 and recommended a general application of antibiotics (in contrast to the currently applicable guidelines in Germany), while amoxicillin was considered as substance of choice. 92.7% of the children actually received amoxicillin, 13% in combination with a beta-lactamase inhibitor. However, in 85% underdosage and in 3.4% overdosage was observed. Regarding the duration of treatment (for children older than 6 years, a duration of 10–14 days was recommended!), the prescriptions were in accordance to the guidelines only in 50.7% of the cases. Overall, only 17 of 207 children (8.2%) were treated completely in conformity to the guideline. Experienced physicians (defined as 20 or more years of professional activity) deviated more often from the guidelines than younger physicians (OR=6.49; [1.71;24.66], p=0.006).

A study of Stapleton and Mills [68] about the diagnostics of Menière’s disease only marginally touches the adherence to current guidelines. The authors compared the diagnostic criteria of the AAO-HNS Committee on Hearing and Equilibrium Guidelines with the criteria of the original publication of Prosper Menière from 1861. Based on 650 cases, it became obvious that the AAO-HNS criteria led 3 times more frequently to the diagnosis of (possible or probable) Menière’s disease compared to the original criteria. But the authors also state that this fact would not lead to modifications of the treatment scheme. 

To complete, it must also be mentioned that the symptoms of Menière’s disease and vestibular migraine overlap and that both diseases have to be considered in the differential diagnosis because the therapeutic procedures are different. Of course, Prosper Menière was not aware of this aspect.

Further, a recent study on prophylaxis of thromboembolism in otolaryngology from England is available [42]. The authors refer to a NICE guideline from 2010. They cite retrospectively assessed data, while 0.11–0.3% of the ENT-specific inpatients had venous thromboembolism. The authors indicate a probably much higher incidence because of asymptomatic events. The prospective study design consisted of 2 audits with (poster) presentation in an ENT department performed in-between. While only in 13 of 23 surgical cases a risk assessment was conducted in conformity to the guidelines in the first cycle, it was made in 26 of 27 cases (p<0.0001) after intervention. This study shows the benefit of on-site intervention, however, there are no data on how long the adherence to the guideline continued.

## 11 Medical education

The obligation to medical education of physicians is regulated in the law on medical practice and the education regulations of the regional medical associations. In general, the regulations of those medical associations are based on the recommendations of the Federal Medical Association on medical education [16]. Medical education is a professional obligation. This obligation of panel physicians expects 250 CME credits in 5 years, a specialist working in a hospital needs 150 CME credits in 3 years. Residents fulfil their obligation to education based on the educational curriculum and thus do not need to collect CME credits. The regulations mention different possibilities of educational measures, certain requirements (e.g. regularly reading specific journals) do not exist. However, it is recommended to be regularly informed about the “official” publications (in Germany: Deutsches Ärzteblatt, publications of the respective regional medical associations) because they also contain information about modifications of relevant requirements. 

This means that in the course of time the specialist standard is also subject to changes. 

It may be expected that the proof of fulfilled medical education will play a key role in future physician liability processes. 

The non-fulfilment of this obligation is considered as severe violation of the medical obligations, accordingly reductions of the medical honorary are justified. For hospital physicians, a violation of this educational obligation will also lead to penalties of the employer. 

Since the Deutsche Ärzteblatt, the journals of the regional medical associations, and the ENT-specific journals regularly offer CME projects, the collection of a sufficient number of CME credits is easy to perform for every physician. 

The proof of CME credits is currently the only way to prove the participation in educational measures according to the requirements. Studying the specific literature (books, journals) cannot be proven even if respective receipts for purchases can be presented. Anyhow, private studies are automatically taken into account of the CME crediting. 

The work group LA-MED Kommunikationsforschung im Gesundheitswesen e.V. (communication research in healthcare) investigated the coverage of medical publications for different disciplines, among others also for ENT specialists (private practices, chief physicians and senior physicians in hospitals). Only the following titles were considered (number of prints in parentheses).

- Forum Hals-, Nasen-, Ohrenheilkunde: Omnimed-Verlag, mainly distributed free of charge (6,500)
- HNO: Springer Publication, mainly paid subscriptions (1,800)
- HNO-Mitteilungen: Deutscher Ärzte-Verlag, automatic subscription in cases of membership in the Professional Association (5,400)
- HNO-Nachrichten: Springer Publication, mainly distributed free of charge (5,200)
- Deutsches Ärzteblatt: Deutscher Ärzte-Verlag, automatic subscription as member of the medial association (303,000)

**Figure 3:**
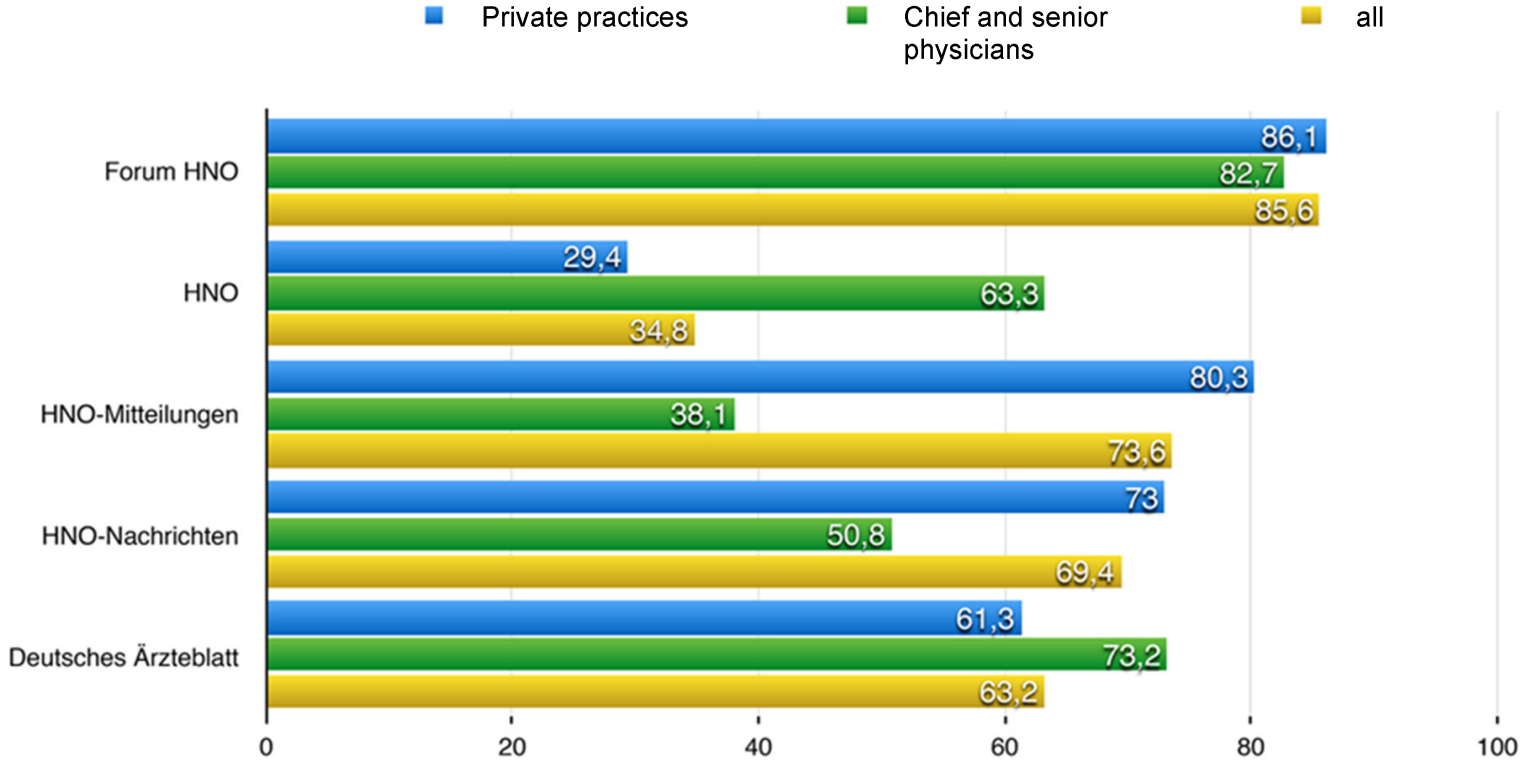
Coverage of 5 German medical journals – number of readers per issue in % (source: www.la-med.de)

Figure 3 (Fig. 3) shows the distribution of those 5 mentioned journals, based on the number of readers per issue. It becomes obvious that especially ENT physicians in practices do not receive the journal with the highest scientific impact (HNO). For notification and finally implementation of guidelines regarding ENT practitioners, the publication in the journal “HNO” is obviously not sufficient. The second German ENT-specific journal (Laryngo-Rhino-Otologie) was not included in the above-mentioned investigation. In analogy, it may be assumed that the number of readers per issue is similar to the value of the journal “HNO”. The journal “HNO-Mitteilungen” reaches in particular ENT practitioners but only to a limited extent hospitals. The journal “Deutsches Ärzteblatt” reaches both groups to a comparable percentage, but it is doubted that it publishes all guidelines of all specific societies. 

The other 2 journals are rather editorially and commercially oriented. 

It must remain open to what extent residents in hospitals use the mentioned print media. 

Regarding the discipline of urology, this study revealed that 93.5% of the chief and senior physicians as well as 78.4% of practicing urologists (=83.3% of all investigated urologists) read the journal “Der Urologe”, which is a fee-based subscription. 

So it is a definite obstacle for the implementation of guidelines in medical routine that the target group(s) that should receive a certain publication in print media are incompletely reached. 

In other words: currently there is no print medium that reliably reaches all otolaryngologists (including residents) in practices and hospitals. 

Now the question must be asked to what extent otolaryngologists use information from the internet in order to be up-to-date regarding guidelines. The access to the guideline platform of the AWMF is possible without registration so that specific allocation of downloads is not possible. However, the AWMF publishes a top 25 list of the downloaded guidelines. With more than 610,000 downloads, the guideline on iron deficiency anemia ranks first on the hit list of October 2015; a guideline of the German Society of Oto-Rhino-Laryngology, Head and Neck Surgery, was not found among those top 25 guidelines. The guideline on idiopathic facial paresis of the German Society of Neurology ranked 12^th^. 

It cannot be determined how many (ENT) physicians keep informed about current guidelines by accessing the AWMF website. 

Also other websites or platforms allow an access to current guidelines. The best known is probably the platform of coliquio. Via http://www.aezq.de/ the National Disease Management Guidelines and further information on the methods of guidelines are found. 

Furthermore, it is recommended to access the guidelines not only on the screen but to download and safe them on the own computer. This recommendation is based on the fact that the AWMF deletes guidelines from its website that are no longer valid. Of course, this avoids the accidental access to expired guidelines and their application, but it also makes it impossible to identify the status of guideline recommendations for a certain time in the past. This aspect may be important if at a later date a justification for a certain procedure has to be provided by referring to a specific guideline. If necessary, the expired guideline can be ordered at the specific society. 

## 12 Conclusion

Since meanwhile 20 years, the concept of evidence-based medicine is known in Germany. The implementation of this concept was and is part of a quality initiative in healthcare that is based on specific laws and other requirements that are continuously modified in the course of time. Since then, much manpower and also many financial resources were invested in the development of guidelines; due to the initiative of the AWMF, this development process is meanwhile mostly standardized. In contrast, the implementation of those guidelines in medical routine, in particular in the discipline of otolaryngology, is still insufficient. Muche-Borowski et al. [51] used the term of evidence-practice gap. 

The present article intended to describe the – manifold – reasons for those discrepancies and at the same time encourage higher adherence to guidelines. Hereby, guidelines must not be understood as unnecessary paternalism or regimentation, but as scientifically and medically justified recommendation that shall enrich and not impair medical action. 

However, it must also be stated that currently no medium is available that keeps all otolaryngologists (in hospitals and practices) as well as all residents reliably informed about new guidelines. 

## Used websites

- http://www.awmf.org/
- http://www.choosingwisely.org/
- http://www.cochrane.org/
- https://www.coliquio.de/
- http://www.g-i-n.net/
- http://www.leitlinien.de/
- http://ebm-netzwerk.de/

## Notes

### Conflict of interest

The author declares that he has no competing interest.

## Figures and Tables

**Table 1:**
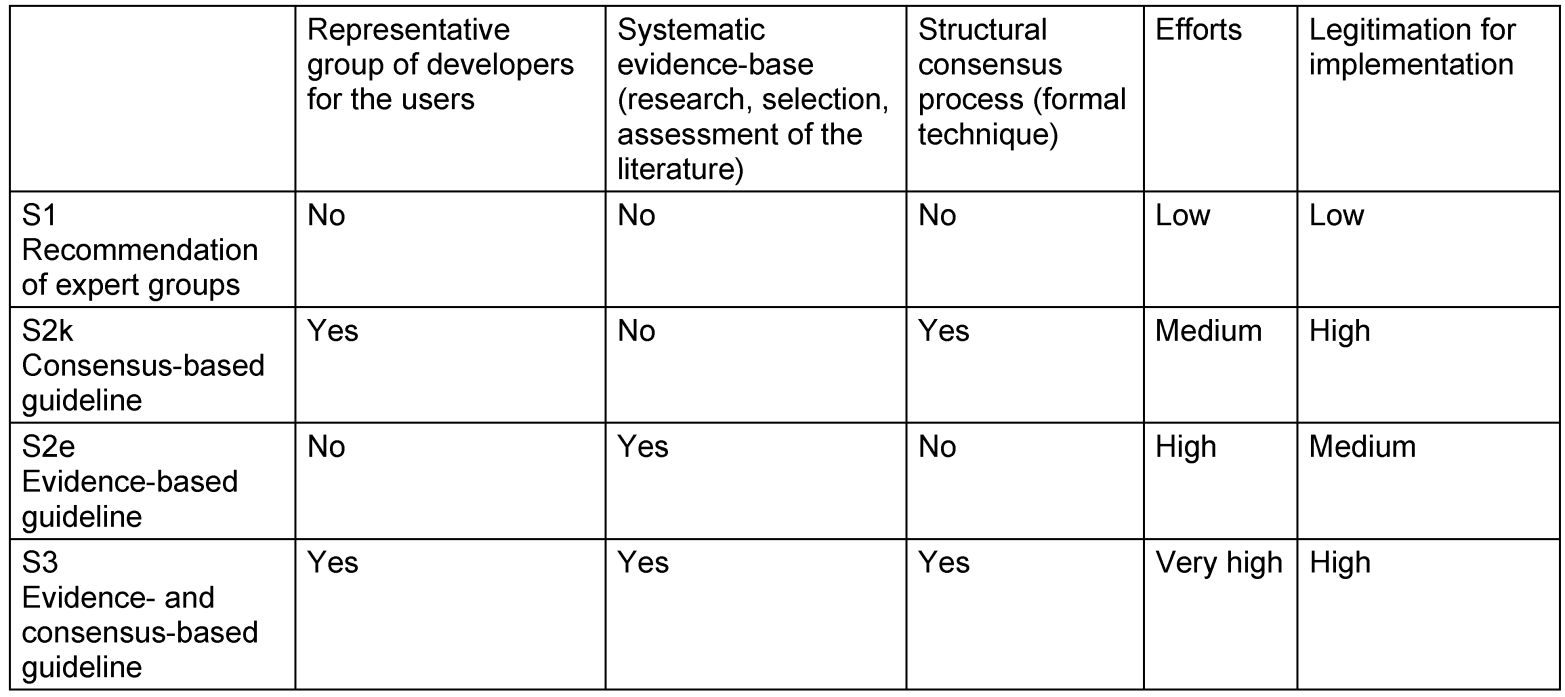
Methodical quality of guidelines: classification in stages of the AWMF (modified according to Follmann, Kopp, and Selbmann)

**Table 2:**
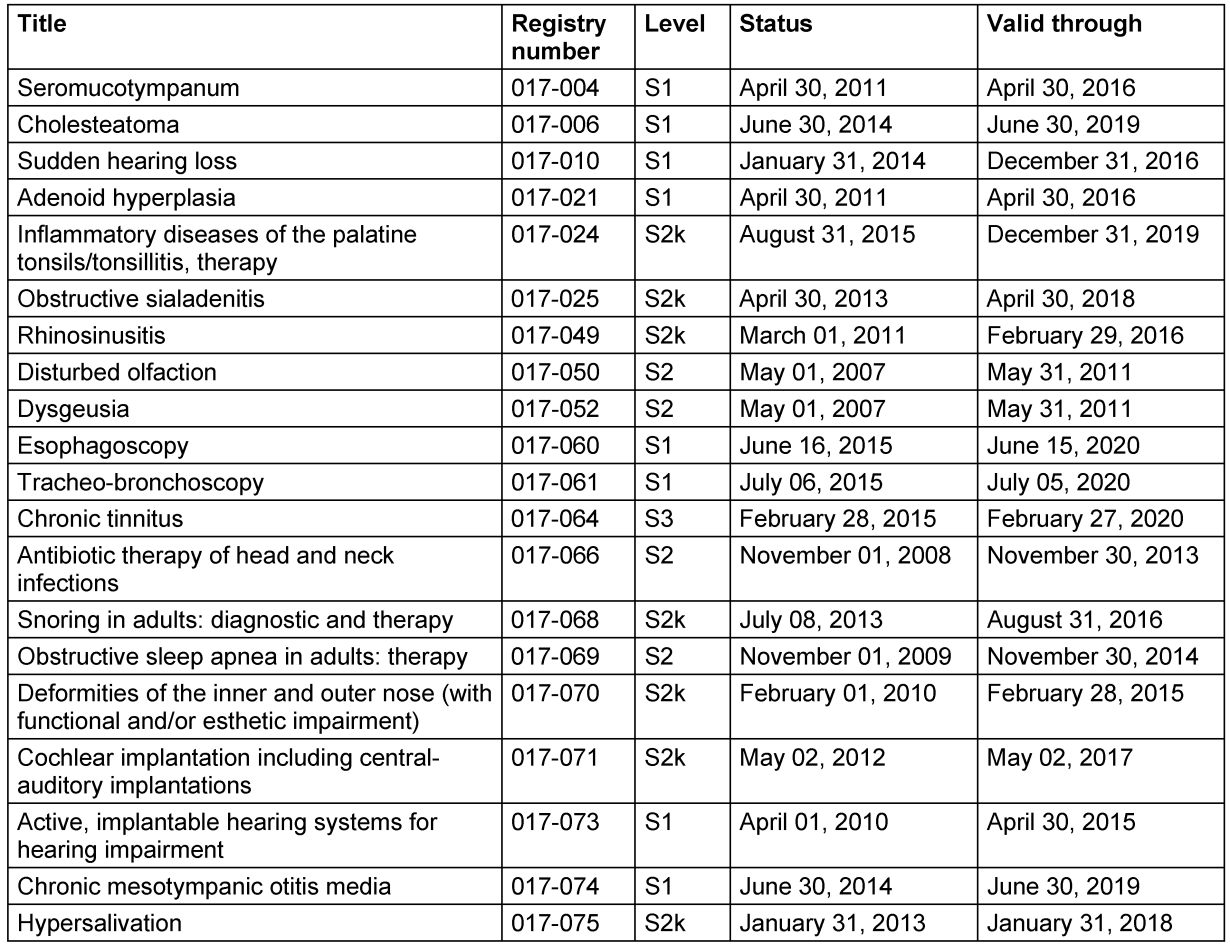
Current AWMF guidelines of the German Society of Oto-Rhino-Laryngology, Head and Neck Surgery (as of September 30, 2015, accessible via www.awmf.org)

**Table 3:**
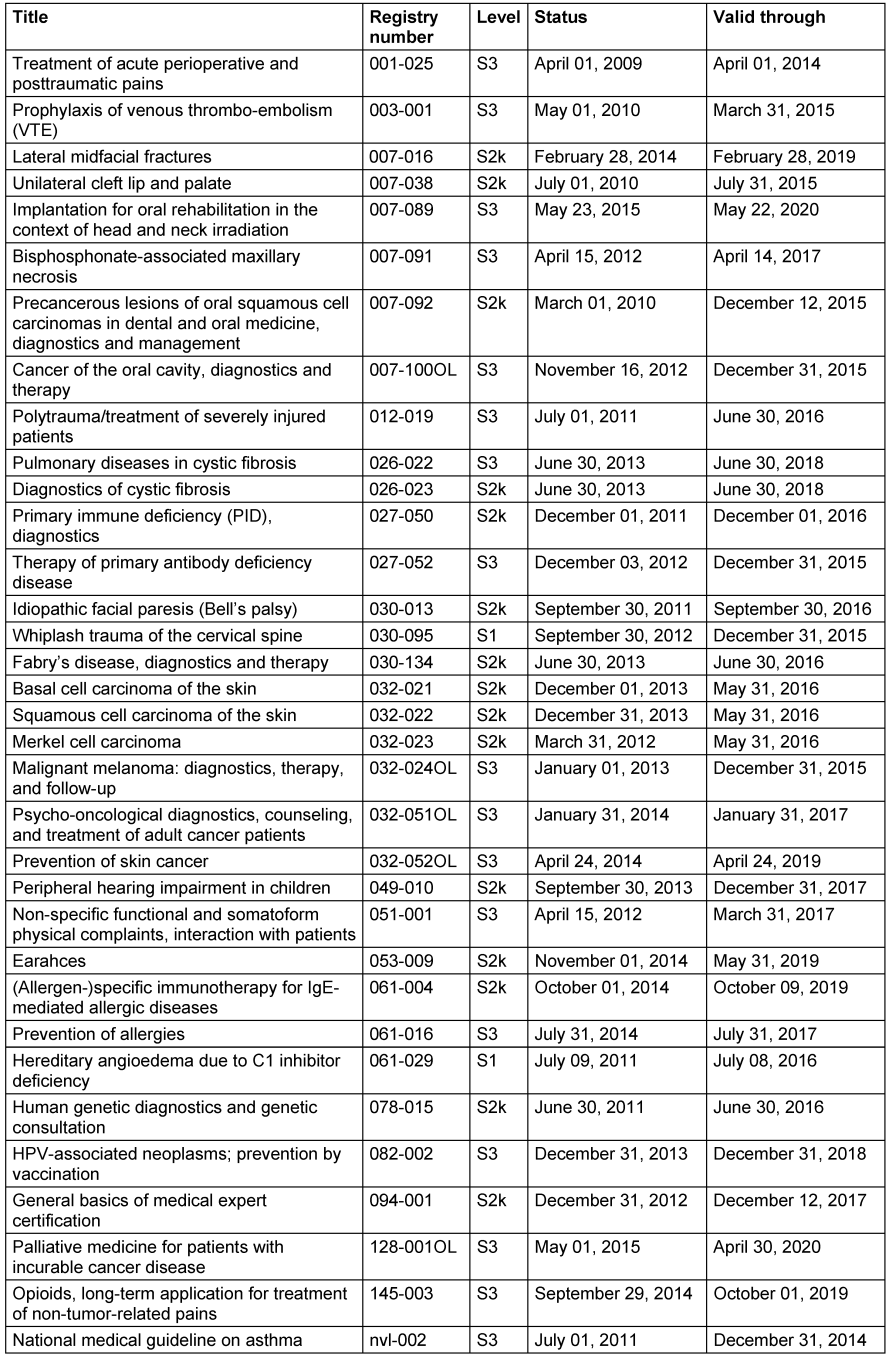
Current AWMF guidelines established with contribution of the German Society of Oto-Rhino-Laryngology, Head and Neck Surgery (as of September 30, 2015, accessible via www.awmf.org)

**Table 4:**
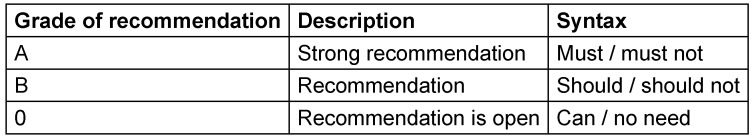
Graduation of the recommendations (AWMF regulations of 2012)

**Table 5:**
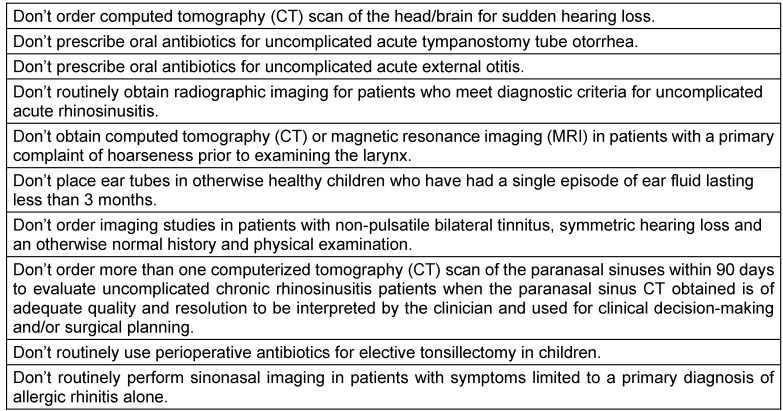
10 statements of the choosing wisely campaign for otolaryngology

**Table 6:**
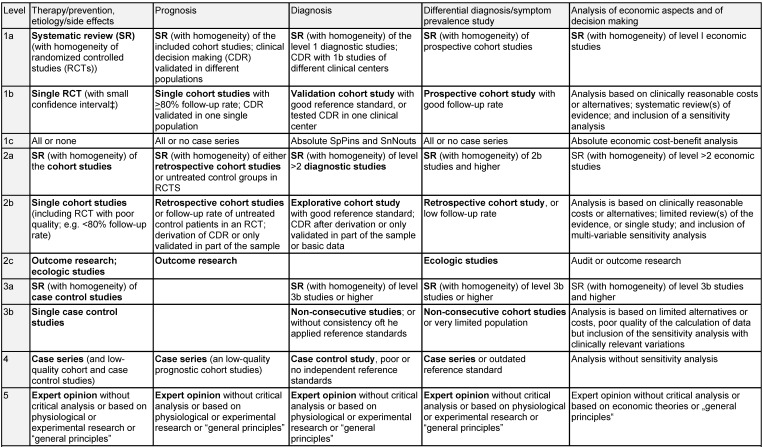
Oxford Centre for Evidence-based Medicine – levels of evidence (March 2009) [54]

**Table 7:**
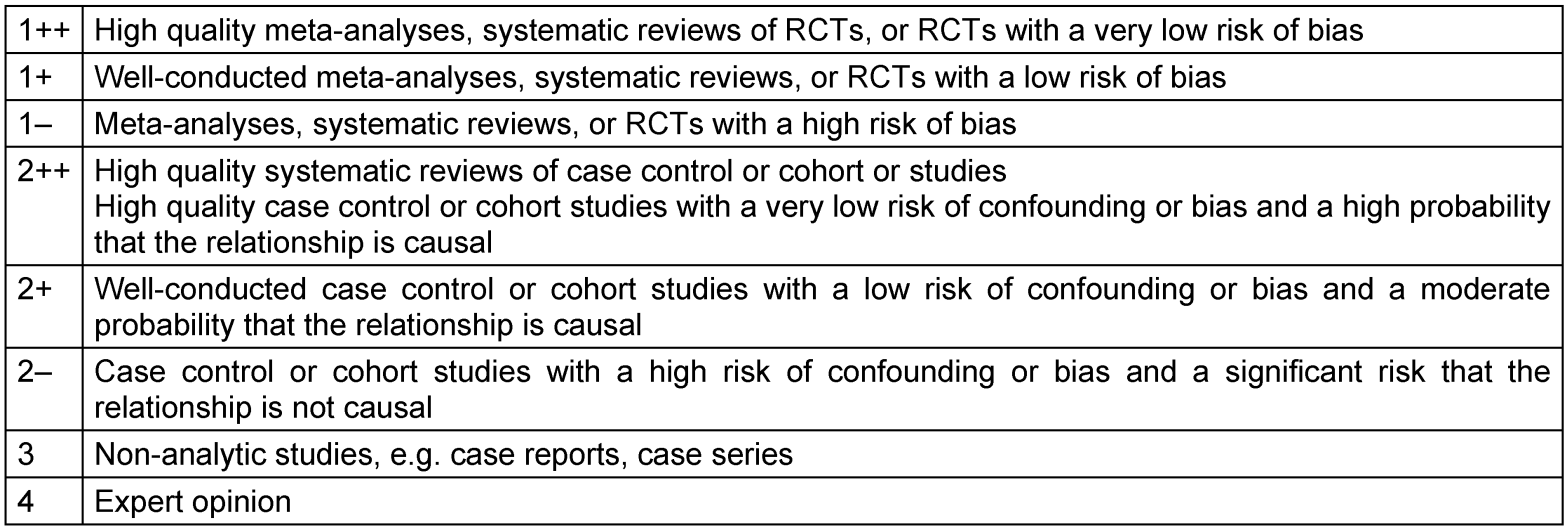
SIGN levels of evidence (Scottish Intercollegiate Guidelines Network)

**Table 8:**
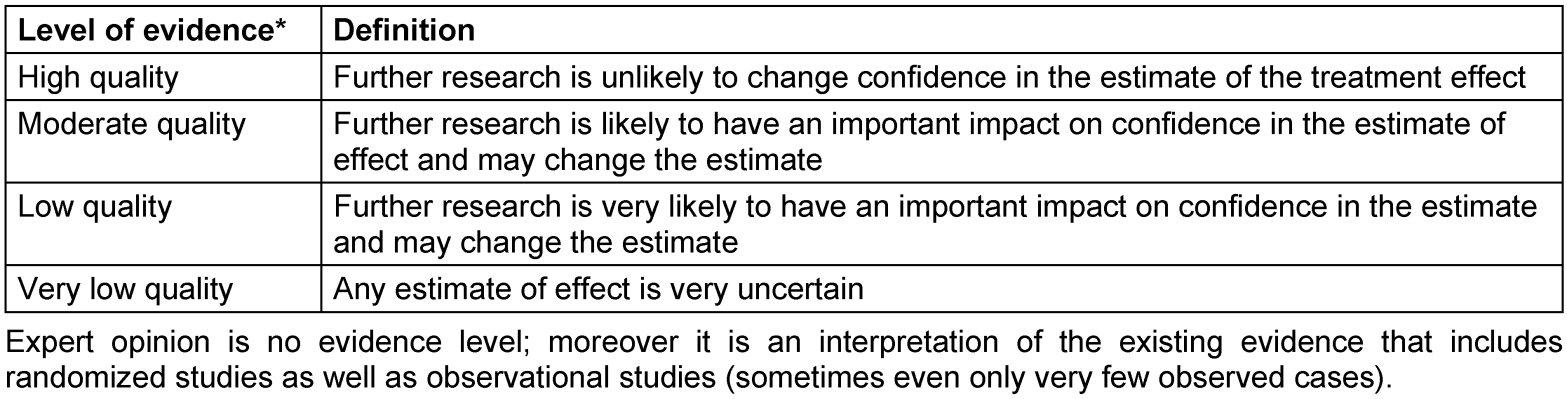
GRADE: Grading of recommendations assessment, developing, and evaluation [62]
